# Wild and domesticated *Moringa oleifera* differ in taste, glucosinolate composition, and antioxidant potential, but not myrosinase activity or protein content

**DOI:** 10.1038/s41598-018-26059-3

**Published:** 2018-05-22

**Authors:** Gwen M. Chodur, Mark E. Olson, Kristina L. Wade, Katherine K. Stephenson, Wasif Nouman, Garima, Jed W. Fahey

**Affiliations:** 1 Cullman Chemoprotection Center, Johns Hopkins University, Baltimore, Maryland USA; 2 Johns Hopkins University Bloomberg School of Public Health, Department of International Health, Center for Human Nutrition, Baltimore, Maryland USA; 3 Instituto de Biología, Universidad Nacional Autónoma de México, Tercer Circuito de Ciudad Universitaria, Ciudad de México, 04510, Mexico; 4 The International Moringa Germplasm Collection, Ejido de la Reforma Agraria, Jalisco, Mexico; 5 Johns Hopkins University School of Medicine, Department of Pharmacology and Molecular Sciences, Baltimore, Maryland USA; 6 Department of Forestry, Range, and Wildlife Management, Bahauddin Zakariya University, Multan, Pakistan; 7 Department of Botany, University of Delhi, Delhi, 110007 India; 8 Johns Hopkins University School of Medicine, Department of Medicine, Division of Clinical Pharmacology, Baltimore, Maryland USA; 9 Present Address: Graduate Group in Nutritional Biology, UC Davis, Davis, California, USA

## Abstract

Taste drives consumption of foods. The tropical tree *Moringa oleifera* is grown worldwide as a protein-rich leafy vegetable and for the medicinal value of its phytochemicals, in particular its glucosinolates, which can lead to a pronounced harsh taste. All studies to date have examined only cultivated, domestic variants, meaning that potentially useful variation in wild type plants has been overlooked. We examine whether domesticated and wild type *M*. *oleifera* differ in myrosinase or glucosinolate levels, and whether these different levels impact taste in ways that could affect consumption. We assessed taste and measured levels of protein, glucosinolate, myrosinase content, and direct antioxidant activity of the leaves of 36 *M*. *oleifera* accessions grown in a common garden. Taste tests readily highlighted differences between wild type and domesticated *M*. *oleifera*. There were differences in direct antioxidant potential, but not in myrosinase activity or protein quantity. However, these two populations were readily separated based solely upon their proportions of the two predominant glucosinolates (glucomoringin and glucosoonjnain). This study demonstrates substantial variation in glucosinolate composition within *M*. *oleifera*. The domestication of *M*. *oleifera* appears to have involved increases in levels of glucomoringin and substantial reduction of glucosoonjnain, with marked changes in taste.

## Introduction

Understanding the ways that economically important domesticated plants differ from their wild ancestors is essential for maximizing the benefits of domesticated plants to humanity^1^. Wild ancestors represent a vast storehouse of genetic resources with proven potential for addressing shortcomings of important crops^2,3^. Understanding the phenotypic starting point of domesticated plants is essential for identifying the most important changes that plants have undergone under domestication and therefore for preserving and accentuating these traits. Here, we provide a vivid example of how domestication appears to have radically altered the phytochemical profile of a nutritionally and medicinally important tree that is cultivated across the tropics. Our results identify a situation in which the change in the profile of phytochemicals of interest in the domesticate with respect to the wild ancestor has been so radical that, at least with respect to these phytochemicals, improvement is probably best accomplished by focusing on domesticated variants rather than wild type ones. *Moringa oleifera* Lam. is a drought-resistant tree native to India and now cultivated in all tropical countries^4,5^. The tree is fast growing, easily reaching 8 m tall in its first year from seed. It grows well in dry tropical localities receiving less than 500 mm of precipitation per year, even when the wet season is short. The leaves of *M*. *oleifera* provide a nutritious leaf vegetable (eaten either fresh or cooked), with 20-30% protein content in the leaflets by dry weight^6^ as well as high quality edible seed oil. *Moringa oleifera* contains substantial amounts of phytochemicals, notably glucosinolates (GS)^7–10^. Investigation into its medicinal properties has provided evidence of antibiotic properties^11,12^, as well as anti-fungal^13^, anti-hyperglycemic^14–16^, anti-hyperlipidemic^17,18^, anti-ulcer^19^, anti-hypertensive^20,21^, and cancer suppressing^22–25^ activities.

Many of these properties in *M*. *oleifera* are plausibly attributed to GS and their cognate isothiocyanates. Numerous studies of isolated *M*. *oleifera* isothiocyanates show activity *in vitro* and in animal systems of antibiotic activity, phase 2 detoxification, antioxidant, and anti-inflammatory response^26–29^, to name a few. We focus upon the GS herein.

To date, all studies of GS in *M*. *oleifera* have focused on domesticated variants across which there are substantial morphological and developmental differences. Domesticated *M*. *oleifera* is an important part of dietary and medicinal traditions throughout India, especially in the south where the immature fruits are consumed daily. From India, the plant has been moved to other parts of the world, including the Philippines, where it is an important leaf vegetable. From the Philippines, sailors brought the plant to the New World on the Manila-Acapulco galleon trade^30^. In general, domesticated *M*. *oleifera* grows very quickly, flowering and fruiting in the first year from seed; has dark green, rounded leaflets; produces fruits with thick, fleshy fruit valves; and has seeds with tough seed coats and dark seeds. The area of wild occurrence of *Moringa oleifera* is mostly in the Indian state of Punjab, extending into the adjacent areas of Himachal Pradesh and a small corner of Haryana State. While the relationship of these plants to domesticated *M. oleifera* is still being investigated, we refer to these plants here as “wild type” *M*. *oleifera*. These plants grow at low elevation in subtropical deciduous forest and differ from the cultivated plants in that they take several years before flowering and fruiting; have pale green, more angular leaflets; produce fruit with thinner, less fleshy fruit valves; and have seeds with soft, spongy seed coats and pale seeds. Wild type plants are commonly cultivated, often to the near exclusion of domestic types, throughout Pakistani Punjab, and the Indian states of Punjab, the lowlands of Himachal Pradesh, and eastward to Bihar (Garima and Olson, unpublished data). Unlike domestic plants, the wild type plants are not part of normal diets, though they are important medicinal plants in local communities.

Our fieldwork in India suggests that there is appreciation of a substantial taste difference between “wild” *M*. *oleifera*, which are often referred to as inedible (Garima and Olson unpublished data), and domestic *M*. *oleifera* plants. It is likely that differences in phytochemical composition underlie this distinction. Taste often strongly directs plant selection^31^, and to the extent that *M*. *oleifera* isothiocyanates underlie taste differences across variants, selection based upon taste could affect its potential medicinal properties.

We ask whether wild versus domestic plants classified based on taste possess similar biological activity or nutritional content. We carried out taste tests using both trained and untrained taste testers to confirm the anecdotal evidence of the wild type’s bitter flavor. Previous work has determined that differences in the pungency of *M*. *oleifera* accessions were not associated with GS content^32^. However, the conversion of stable GS to reactive isothiocyanates accounts for the pungent taste of plants that contain them, and this conversion is dependent on myrosinase^33–36^. It may be that differences in amount, specificity, or specific activity of myrosinase, rather than the amounts of its substrate GS, are responsible for different pungencies^32^. Phytochemical content, and therefore taste, can strongly affect frequency of consumption^37–41^, and in turn, frequency of consumption affects phytochemical dose and the benefits that accrue from them. As an index of direct antioxidant activity, we use ABTS^•+^ 2,2′-azinobis(3-ethylbenzthiazoline-6-sulfonic acid) to measure the ability the endogenous antioxidants present in a leaf homogenate, to scavenge free radicals. We ask whether there is variation in ATBS^•+^ response between wild type and domestic cultivars.

Finally, we evaluate protein content in both wild type and domesticated plants. The very high protein content of *M*. *oleifera* leaflets makes this drought-resistant plant an extraordinarily attractive tool for addressing dietary protein deficiency throughout the tropics. Selection on taste, GS content, or any other factor could potentially affect protein content. Because variants that simultaneously maximize protein content together with nutraceutical effect would be maximally desirable, it is essential to understand whether protein and GS content appear related to one another negatively or positively, or whether their levels appear uncoupled.

We predicted that wild type and domestic *M*. *oleifera* would contain different quantities of myrosinase or GS, and/or differentially active myrosinase, and/or a substantial difference in GS profile. This variation in GS profiles should be associated with differences in indirect antioxidant activity, but protein levels should vary independently of GS levels. To test the hypothesis that acceptability from a taste perspective is inversely correlated with myrosinase activity, we examined protein, GS, and myrosinase content, as well as direct antioxidant activity (potentially a function of flavonoid levels or other features of each accession’s unique biochemistry) of 36 accessions of *M*. *oleifera* grown in the same location and harvested at the same time.

## Results

### Validation of Drying Method

Myrosinase activity was fully preserved by silica gel drying of the leaves, as was antioxidant potential; GS content was, in fact, somewhat higher when normalized per fresh weight, compared to fresh harvested or freeze-dried samples, suggesting that some hydrolysis occurred even with careful handling and drying (data not shown). Based upon these results, for the analyses reported herein, we employed a drying process at the rural *Moringa* germplasm collection site in which leaves were harvested, immediately placed in coin envelopes, and sealed in vessels containing activated silica gel. They were sent to the USA for analysis when fully dry.

### Total Protein Content

**Figure 1:**
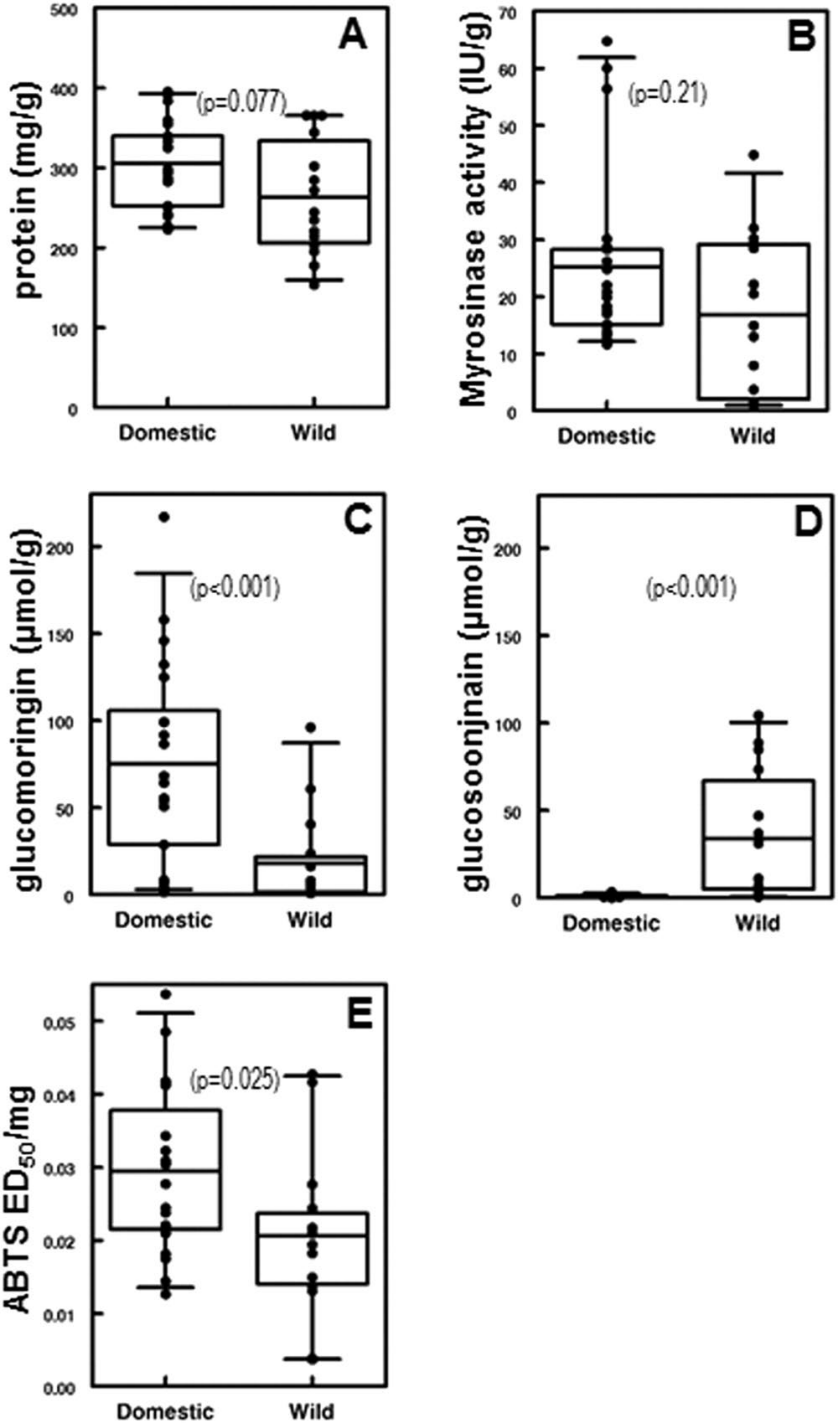
Differences between domestic and wild type *Moringa oleifera*. Significant differences were determined by Mann Whitney test. (**A**) Protein content (U = 213, p = 0.077); (**B**) myrosinase activity (U = 197, p = 0.21); glucosinolate content for (**C**) glucomoringin (U = 266, p < 0.001), (**D**) glucosoonjnain (U = 26.5, p < 0.001); and (**E**) direct antioxidant activity (U = 217, p = 0.025).

Mean per-individual protein content varied from 15.37 to 39.52% (153.7 to 395.2 mg/g; Table 1). Mean protein content of domesticated *M*. *oleifera* accessions was 30.24% (95% CI 27.85–32.63%). Mean protein content of wild type *M*. *oleifera* accessions was 26.28% (95% CI 22.28–30.27%). As shown in Fig. 1A, there was no significant difference in protein content between wild type and domestic variants of *M*. *oleifera* (Mann-Whitney U = 213, p = 0.077).

**Table 1:** Provenance of plants grown and harvested in Jalisco MX, and the results of analytical tests run on their dried leaves.

Plant ID	Wild/Domestic	Locality of original collection	Myrosinase activity (IU/g)	Protein (mg/g)	Glucosinolate Content		Antioxidant Activity
					4RBGS (µm/g)	4GBGS (µm/g)	ED50 (TE/mg)
1	domestic	Tolagnaro, Madagascar	18.32	239.48	55.57	0.78	0.022
2	domestic	Mérida, Yucatán, Mexico	20.75	324.71	145.90	0.99	0.018
4	domestic	Isiolo, Kenya	13.32	395.18	157.95	0.88	0.054
6	domestic	Superfoods Moringa for Life, Thailand, as PKM cultivar, but has short fruits, unlike PKM	12.53	298.2	91.81	0.96	0.049
9	domestic	Isiolo, Kenya	64.73	359.4	1.53	0.8	0.031
13	domestic	Isiolo, Kenya	60.01	334.66	6.71	0.61	0.042
16	wild	Jhang, Punjab, Pakistan	1.04	244.48	6.26	37.01	0.021
17	wild	Qila Didar Singh, Punjab, Pakistan	20.52	220.65	1.84	88.65	0.043
27	wild	Khair Pur, Sindh Provice, Pakistan	29.39	203.5	8.20	84.82	0.022
52	domestic	Voi, Kenya	15.19	286.61	99.25	1.37	0.041
57	wild	Khair Pur, Sindh Provice, Pakistan	44.83	365.39	60.82	5.99	0.014
61	wild	Qila Didar Singh, Punjab, Pakistan	1.46	153.70	0.89	11.18	0.004
66	domestic	street tree, Chrompet, Chennai, Tamil Nadu, India	19.91	284.89	64.3	0.91	0.014
81	domestic	Mesa Garden Nursery, Arizona, USA	17.63	250.54	28.95	1.08	0.03
82	domestic	Ile de la Réunion, Indian Ocean	11.64	241.93	99.03	0.84	0.032
85	domestic	Gerhard Kohres nursery, Germany	56.39	252.34	50.78	1.50	0.024
140	wild	Northern India; Shankar Nursery, Chandigarh, 2000	3.77	284.56	1.71	104.36	0.015
141	wild	Northern India; Shankar Nursery, Chandigarh, 2000	1.19	271.59	0.94	7.43	0.004
142	wild	Northern India; Shankar Nursery, Chandigarh, 2000	30.16	177.59	5.79	73.54	0.028
143	wild	Northern India; Shankar Nursery, Chandigarh, 2000	7.96	234.56	1.07	1.47	0.013
152	wild	Northern India; Shankar Nursery, Chandigarh, 2000	14.96	195.5	4.12	0.59	0.021
171	wild	Qila Didar Singh, Punjab, Pakistan	0.99	301.86	23.68	47.03	0.024
183	domestic	agricultural variant provided by Swaminathan Institute, Chennai, Tamil Nadu, 1998	17.04	390.91	132.27	1.19	0.022
192	wild	Khair Pur, Sindh Provice, Pakistan	22.17	344.22	96.1	6.42	0.019
248	domestic	Paritosh Herbals Nursery, Dehra Dun, Uttarakhand	17.36	292.93	86.62	1.56	0.03
249	domestic	Paritosh Herbals Nursery, Dehra Dun, Uttarakhand, 2014	13.85	282.5	54.30	3.48	0.024
255	wild	Northern India; Shankar Nursery, Chandigarh, 2000	13.02	365.24	40.58	2.43	0.042
256	wild	Northern India; Shankar Nursery, Chandigarh, 2000	28.52	365.39	16.31	31.15	0.021
257	domestic	Nueva Italia, Michoacán, Mexico	26.2	332.82	125	1.53	0.028
279	wild	Northern India; Shankar Nursery, Chandigarh, 2000	32.02	213.47	1.74	4.78	0.018
288	domestic	Paritosh Herbals Nursery, Dehra Dun, Uttarakhand, 2014	24.78	227.35	4.63	0.59	0.018
289	domestic	Paritosh Herbals Nursery, Dehra Dun, Uttarakhand, 2014	28.27	222.64	8.59	0	0.013
299	domestic	Miguel Hidalgo, Jalisco, Mexico	14.83	326.43	216.95	2.17	0.021
PKM	domestic	cultivated in Cihuatlán, Jalisco, Mexico	30.15	339.57	28.7	1.07	
sn MX	domestic		20.88	383.45	53.93	1.55	
sn MX	domestic	PlantzAfrica Nursery, South Africa	22.02	354.7			
sn ZA	domestic		28.73	294.39	68.35	0.61

### Myrosinase Activity

Myrosinase activity ranged from 0.99 to 64.73 I.U./g dry weight (Table 1). Mean myrosinase activity of domesticated cultivars was 25.38 I.U./g (95% CI: 18.24–32.51); mean of the wild type accessions was 16.80 I.U./g (95% CI: 9.00–24.60). As shown in Fig. 1B, there was no significant difference in myrosinase activity between wild type and domestic variants of *M*. *oleifera* (U = 197; p = 0.21).

### Glucosinolates (GS)

**Figure 2:**
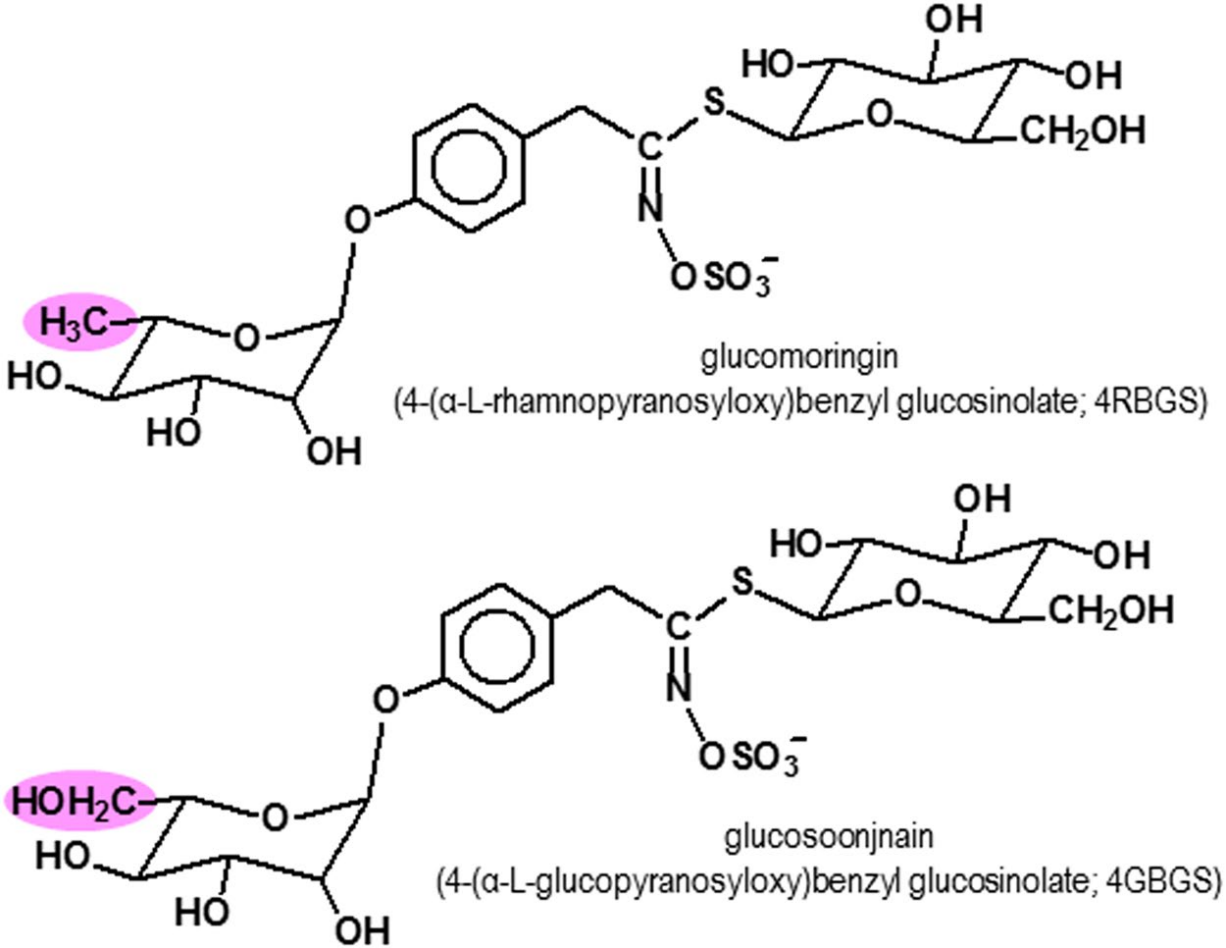
Structures of the two predominant glucosinolates from *Moringa oleifera*. Glucomoringin (4RBGS) and glucosoonjnain (4GBGS).

HPLC chromatograms of wild type and domesticated accessions featured one of two predominant GS peaks (Supplementary Figure S1). One of these was glucomoringin (4-(rhamnopyranosyloxy)benzyl glucosinolate or 4RBGS) (Fig. 2), the GS that has been long associated with *M*. *oleifera*^42^. The second HPLC peak was glucosoonjnain (4-(glucopyranosyloxy)benzyl glucosinolate or 4GBGS), a novel GS shown in Fig. 2 and that is first described in the companion paper to this^26^. These GS and their acetylated derivatives were by far the most abundant in both wild type and cultivated accessions. Other minor GS made up only a small fraction of the total in these samples, and are ignored for purposes of this comparison. There were significant differences between relative levels of the two GS by Mann Whitney. Both GS were present in varying amounts in each sample. We observed strong differences between wild type and domestic variants as follows.

#### Glucomoringin (4RBGS)

Glucomoringin concentrations ranged from 0.89 to 216.95 µmol/g dry weight (Table 1). Mean glucomoringin content of domesticated cultivars was 75.29 µmol/g (95% CI 49.32–101.26). Mean glucomoringin content of wild type accessions was 18.00 µmol/g (95% CI 2.68–33.32). The difference between mean glucomoringin concentrations in a comparison of domesticated and wild type accessions reached statistical significance as shown in Fig. 1C (U = 266, p < 0.001).

#### Glucosoonjnain (4GBGS)

Glucosoonjnain concentrations ranged from 0 to 104.36 µmol/g dry weight (Table 1). Mean glucosoonjnain level in domesticated cultivars was 1.16 µmol/g (95% CI 0.85–1.49). Mean glucosoonjnain in wild type accessions was 33.79 µmol/g (95% CI 13.35–54.23). Wild type accessions had significantly more glucosoonjnain than cultivated accessions (U = 26.5, p < 0.001) as shown in Fig. 1D.

### Antioxidant Potential

Mean antioxidant activity ranged from 0.004 to 0.054 Trolox Equivalents (TE)/mg dry weight (Table 1), with domesticated plants having significantly higher activity. Mean antioxidant activity of domesticated cultivars was 0.030 TE/mg (95% CI 0.024–0.035). Mean antioxidant activity of wild type plants was 0.021 TE/mg (95% CI 0.015–0.027). Wild type and cultivated accessions differed significantly (U = 217, p = 0.025), with domestic plants having higher direct antioxidant activity (Fig. 1E).

### Taste Testing

Agreement between blinded participants’ identification and the classification of the plant samples by degrees of bitterness was good (73.3% agreement; Table 2). The participants were especially accurate in the identification of domesticated plants as non-bitter. The probability of correctly identifying a plant by chance alone was high given the dichotomous classification system used; therefore, a Kappa statistic was computed to account for agreement due to chance alone, and it (κ = 0.47), suggests moderate agreement between the subjects’ taste assessment and classification by cultivation status, using established standards of interpretation^43^.

**Table 2:** Results of Blind Taste Tests of Wild type and Domesticated Accessions of *M*. *oleifera*.

Participant taste Identification^1^	Plant cultivation^2^	
	Wild type accessions	Domesticated accessions
Bitter	19	5
Not Bitter	11	25
Total	30	30

^1^Participants assigned blinded samples to categories of “bitter” and “non-bitter” after tasting exemplar accessions.

^2^Wild or domesticated *M*. *oleifera*.

A highly trained sensory analyst (Gail V. Civille, Sensory Spectrum, Inc) provided a technical description of representative leaf samples from both domestic and wild type accessions. Domesticated *M*. *oleifera*, was described as mild tasting, reminiscent of green triplal, fresh, stemmy, green beans, tulip stems, and with a more intense taste when ground between teeth. Wild type *M*. *oleifera* was characterized as reminiscent of green, heavy, leafy - kale, tree leaves, with a low aromatic component.

## Discussion

This study is the first, to our knowledge, to demonstrate substantial differences in GS composition within the tropical tree *Moringa oleifera*. These differences were revealed by including samples from the putative wild ancestral type of *M*. *oleifera*. Our results very clearly separated wild type and domestic populations based solely upon their proportions of the two predominant GS (glucomoringin and glucosoonjnain), with domestic *M*. *oleifera* having high levels of glucomoringin and low levels of glucosoonjnain, and wild type *M*. *oleifera* having low levels of glucomoringin and moderate to high levels of glucosoonjnain. This finding suggests that GS profile and levels are responsive to selection in *M*. *oleifera* and that the relative levels of these compounds could be altered under future breeding. It also seems possible that glucosoonjnain is selected against because of the taste of its cognate isothiocyanates.

Taste differences between wild type and domesticated accessions of *M*. *oleifera* were readily identifiable to both trained and untrained taste-testers. These results support the anecdotal evidence that not all accessions of *M*. *oleifera* are acceptable food sources among communities in which they are grown. In India, wild type *M*. *oleifera* are used mostly for medicine, whereas domestic types are used for both food and medicine.

The differences in taste are potentially caused by the differences in GS profiles between wild type and domestic variants. Our results strongly suggest that domestication of *Moringa* may have selected against glucosoonjnain. Glucosoonjnain is a newly identified GS that we have just described^26^. Unlike glucomoringin (the GS that has been the most commonly described GS in *M*. *oleifera*), glucosoonjnain contains a glucopyranosyloxy-benzyl moiety as its R-group. This difference has implications in terms of reactivity, lipophilicity, electrophilicity, partition coefficients, and all of the chemical and biological properties of GS and their isothiocyanates that are the hallmarks of this large group of compounds^34,44–46^, including taste^47^. We have noted^26^ that glucomoringin induced phase 2 enzyme activation in mammals more potently than glucosoonjnain. If these results translate to potency of induction in humans, then selection favoring medicinal activity would favor higher levels of glucomoringin. It is therefore possible that the process of domestication favored better tasting variants, which happened also to have greater phase 2 induction potential. Overall, these results suggest that the novel GS glucosoonjnain is well-correlated with taste in wild type accessions of *Moringa oleifera* and present only at very low levels in domesticated variants.

Alternatively, it is possible that myrosinase preferentially hydrolyzes glucosoonjnain to its isothiocyanate metabolites, resulting in larger quantities of isothiocyanates with a more intense taste. The isothiocyanate hydrolysis products of GS cleavage have been shown to have potent pharmacological activity in general^34^, as have, specifically, the products of myrosinase action on glucomoringin^48–50^. Future research should compare the metabolomic and medicinal effects of both glucomoringin and glucosoonjnain. Although this study was not powered to address whether myrosinase content may also be responsible for differences in taste between cultivars, there appear to be no significant such differences in myrosinase activity.

As with levels of myrosinase activity, despite marked alteration in glucosinolate profiles, the process of domestication does not appear to have affected protein content. Soluble protein content determined in this study was not significantly different (30.2% and 26.3% for domesticated and wild type accessions, respectively). These percentages are consistent with previous findings, which typically range from 20–30% of leaf dry weight^6,9,51,52^. These results suggest that selective breeding to alter *M*. *oleifera* glucosinolate profiles can be achieved without altering the high protein nutritional value of *Moringa* leaves.

Together with much higher levels of glucomoringin, we found significantly higher direct antioxidant activity in domesticated as compared to wild type plants using the ABTS^•+^ assay. This assay provides an *in-vitro* measure of antioxidant activity, and does not parse the differential action of individual phytochemical components. *Moringa* spp. contain abundant vitamin C and polyphenols, compounds which are expected to have direct antioxidant capacity *in vitro*. Since all plants contain direct antioxidants, results of this assay would require clinical validation to have any human health relevance. It is well known that neither the GS nor their cognate isothiocyanates have such direct antioxidant activity, but rather, isothiocyanates are superior and potent indirect antioxidants^53–58^. In other words, they are remarkably potent inducers of cytoprotective enzymes including a variety of antioxidant enzymes. Although our investigation focused on GS, the observed difference in ABTS^•+^ suggests that in addition to the differential levels of glucomoringin and glucosoonjnain, wild and domesticated *Moringa* cultivars may differ substantially in their content of other classic antioxidants as well.

From a methodological standpoint, we showed that both myrosinase activity and direct antioxidant potential (ABTS^•+^) are preserved by drying fresh *Moringa* leaves in activated silica gel. This is a very low cost method routinely used to collect samples for DNA extraction. It is a method of sample preservation that greatly simplifies collection of tissues and it has permitted us to harvest and ship material from an area where a lyophilizer was not available without fear of spoilage or loss of sample integrity.

In exploring variation in GS profiles across cultivated and wild type *Moringa oleifera*, a strength of this study was that all plants were grown at one location and harvested and dried at the same time, using a method that we validated to show preservation of myrosinase activity. This strongly suggests that differences shown in this analysis are likely the result of true variation between wild and domestic *M*. *oleifera* and not an artifact of soil conditions, environmental exposures, processing method, or sample transit time.

*Moringa oleifera* illustrates the general principle that the wild ancestors of domesticated organisms provide much wider ranges of variation than the domesticates. Here, we show that GS profile varies conspicuously between the putative wild type and the domesticate. With bitter tasting wild types having high levels of glucosoonjnain, and domestic ones having low levels, it is plausible that this difference underlies the strong taste difference between wild type and domestic plants. In turn, this taste difference seems likely to motivate the traditional distinction between the wild type as medicinal but inedible and the domesticate as eminently edible as well as medicinal. Additional research to describe the roles of both glucomoringin and glucosoonjnain may help to elucidate additional mechanisms through which *Moringa oleifera* exerts beneficial health effects, and how it made its way from a wild ancestor, widely regarded as non-edible, to an esteemed food plant with improved medicinal properties.

## Materials and Methods

All methods were performed in accordance with the relevant guidelines and regulations. If not otherwise specified, reagents were purchased from Sigma-Aldrich (St Louis, MO, USA).

### Validation of Drying Method

To confirm that silica gel drying could be used for preserving myrosinase activity in dried leaf samples, a validity study was conducted in Baltimore. *Moringa oleifera* seeds were obtained from Horti Nursery Networks (Erode, Tamil Nadu, India) and planted in Foxfarm^®^ Seed Starter Grow Medium (Arcata, CA, USA) in illuminated flats. After 31 days, fresh leaves were harvested from the young plants and separated into three groups. Fresh leaves were analyzed for myrosinase activity, protein content, GS composition, and antioxidant activity. Remaining leaves were placed into separate envelopes: half were freeze dried while the other half were placed into activated silica gel desiccant (Type II 3.5 mm bead size). Methanolic and aqueous extracts of these two dried sets of samples were analyzed similarly to the fresh leaves.

### Sample Collection and Preparation

Leaves were harvested from trees at the International Moringa Germplasm Collection in Jalisco, Mexico. Provenance and accession numbers of trees sampled are provided in Table 1. Domesticated variants represented the span from agricultural variants that are used in commercial production of moringa products, to variants cultivated as street trees in India, Mexico, Reunion Island, and Kenya. Plants cultivated as street trees seem likely to vary more than variants used for agricultural production, which would be expected to be subject to selection for uniform yield and characteristics. Moreover, we included individuals grown from seed purchased from commercial nurseries in the US, Germany, South Africa, India, and Thailand, because these represent readily available germplasm that many projects focusing on *M*. *oleifera* will obtain. The trees were cultivated under uniform dry tropical conditions on local soil derived from decomposed granodiorite. The collection is subject to a prolonged dry season from November to mid-July, punctuated by a short rainy season. Annual average rainfall is 752 +/− 256 mm, most of which falls as the result of the passage of hurricanes along the coast. Mean annual temperature is 24.9 °C +/− 14.8 to 32 °C^59^.

Individual leaves were removed from 36 (21 domestic and 15 wild type) accessions of *Moringa oleifera*, selecting the uppermost fully developed leaves growing on sun-exposed shoots occurring mid-canopy. The leaves were dried in silica gel upon collection and shipped them to Baltimore. Upon receipt, the samples were lyophilized to guard against incomplete field drying before leaflets were individually removed from the petiolules. The dried leaflets were weighed and used to prepare aqueous (n = 36) or 80% methanol extractions (n = 36). The methanol extracts were frozen at −20 °C and homogenized prior to analysis. Aqueous extracts were prepared individually, immediately prior to analysis in order to preserve myrosinase activity since homogenization, crushing, grinding, or bruising plant samples initiates the conversion of GS to isothiocyanate(s).

### Total Protein Content

Filtered aqueous extracts of plant material were used for measurement of total protein content using the widely used colorimetric assay, the Bicinchoninic Acid or BCA method^60^ in a 96-well microtiter plate format^61^. Absorbance at 562 nm (**A**_562nm_) was determined using a SpectraMax Plus plate reader with SoftMax Pro software (Molecular Devices, Sunnyvale, CA, USA). Pierce^®^ BCA Reagent A and albumin standard were from Thermo Scientific (Rockford, IL).

### Myrosinase Activity

Aqueous extracts were analyzed using a chromogenic enzyme assay^35^. Plant extracts (20 mg/mL) homogenized in deionized water containing active myrosinase were combined with an aqueous solution of 500 µM ascorbic acid and 20 mM sodium phosphate buffer (pH 6.0). **A**_227nm_ was measured as a baseline, and then sinigrin (50 µM) was added. Rates of sinigrin disappearance were measured by monitoring reduction in **A**_227nm_ over 3′ using a SpectraMax Plus plate reader with SoftMax Pro software.

### Glucosinolate Concentration

Dried leaves were homogenized in 80% methanol (20 mg leaves/mL), using a Polytron homogenizer directly in glass test tubes. GS content was evaluated by hydrophilic interaction liquid chromatography (HILIC)^62^. Briefly, methanolic extracts were filtered, diluted 10-fold with 30 mM ammonium formate in 70% acetonitrile, and re-filtered. Dilutions were injected onto a 5 µm, 200 Å, PEEK 150 × 4.6 mm ZIC-HILIC column (Sequant, Umea, Sweden) eluted with isocratic 30 mM ammonium formate in 70% acetonitrile, at a flow rate of 0.5 mL/min, and **A**_235nm_ was monitored on a photodiode array detector for glucosinolate peaks, which were compared to standards. Myrosinase digestion was performed on replicate extracts to demonstrate that observed peaks disappeared, confirming their identity as GS.

### Antioxidant Activity

As a rough proxy measurement of phenolic compounds and vitamin C known to be present in *M*. *oleifera*, we measured direct antioxidant activity. Antioxidant activity of 80% methanolic extracts was assessed using the ABTS^•+^ radical cation decolorization assay^63,64^. Briefly, serial 1:1 dilutions of 50 µL of each compound were performed across four rows of a 96-well microtiter plate; 250 µL of 0.08 mM ABTS^•+^ solution was added to each well in the top two rows, and 250 µL of ethanol added to the bottom two rows, to serve as controls. Absorbance readings (**A**_735nm_) were made immediately (Spectramax Plus with SoftMax Pro software) and then continuously for 5′. The 2′ reading was used to calculate the median effective dose (ED_50_) by plotting percent inhibition compared to “no-extract” controls. Results are expressed as the commonly used Trolox (6-hydroxy-2,5,7,8-tetamethylchroman-2-carboxylic acid) Equivalents (TE) per mg dry plant matter (Trolox is a widely used water soluble vitamin E analog that is used as a standard in experiments of this type).

### Taste Analysis

Taste-testing was conducted at the International Moringa Germplasm Collection in Jalisco, Mexico. Work was performed under a qualitative research protocol approved by the JHU Institutional Review Board (#IRB00090394), and with local approval. All work was performed in accordance with relevant guidelines and regulations and written informed consent was obtained from all participants. Fresh leaves were harvested from 8 accessions identified as having the highest and lowest content of the novel glucosinolate characteristic of wild type *M*. *oleifera* recently given the common name “glucosoonjnain”^26^. Uncooked fresh leaves from cultivated and wild type *M*. *oleifera* were presented to the volunteer panel (n = 10) of local residents who were used to eating domesticated moringa. One accession containing high amounts of glucosoonjnain was presented to panelists as an example of a bitter plant, while an accession identified as containing low amounts of glucosoonjnain was presented as a non-bitter plant. After tasting the comparator plants, the panel independently classified each of the six remaining accessions as “bitter” or “not-bitter” based on the perceived comparison to the exemplar accessions. Samples were presented in random order and all participants tasted samples from the same plant simultaneously. Participants rinsed their mouths before continuing to the next sample. Participant responses were then compared to the identity of the plant to calculate concordance.

To examine qualitative differences between domesticated and wild type plants, taste descriptions of representative leaves from both types of plants were prepared by a trained professional sensory analyst from Sensory Spectrum, Inc., USA, who was not familiar with the taste of *M*. *oleifera* (in contrast to those in Mexico who had already been exposed to *M*. *oleifera*).

### Statistical analysis

We calculated per-individual mean values of myrosinase activity (IU/g) and protein content (mg/g) based on the three replicates per sample, and ED_50_ ABTS^•+^/mg dry leaf from the two replicates. Based on per-individual values, we then tested for significant differences in myrosinase activity, protein content, glucomoringin content (µmol/g), glucosoonjnain (µmol/g) content, and ED_50_ between wild type and domestic *Moringa oleifera*. We used Mann-Whitney U tests after checking assumptions and finding non-normal distributions. All analyses were carried out in R v.3.3.1^65^.

## Electronic supplementary material

Supplemental Figure S1
