# Supplementary material for: Wild and domesticated Moringa oleifera differ in taste, glucosinolate composition, and antioxidant potential, but not myrosinase activity or protein content
Source: Sci Rep. 2018 May 22;8:7995. doi: 10.1038/s41598-018-26059-3 (PMC5964143; doi:10.1038/s41598-018-26059-3)

Wild and domesticated *Moringa oleifera* differ in taste, glucosinolate composition, and antioxidant potential, but not myrosinase activity or protein content

Gwen M. Chodur, Mark E. Olson, Kristina L. Wade, Katherine K. Stephenson, Wasif Nouman, Garima, Jed W. Fahey*

*Corresponding Author:

Jed William Fahey, Cullman Chemoprotection Center, 855 N. Wolfe St., Suite 625, Baltimore, Maryland, USA 21209, email: jfahey@jhmi.edu; phone: 410-614-2607

Supplemental Figure S1. **Partial chromatograms showing the disappearance of glucosinolates following myrosinase treatment of leaf extracts of *Moringa oleifera* rich in each of the two major glucosinolates.** Extracts injected on HPLC prior to treatment (**---**), and after about 2 hours treatment with myrosinase (**---**). (**A)** representative wild type *M. oleifera* (#140 in **Table 1**), rich in glucosoonjnain, and (**B)** representative domesticated *M. oleifera* (#2 in **Table 1**), rich in glucomoringin, both of which disappeared completely following enzymatic treatment.

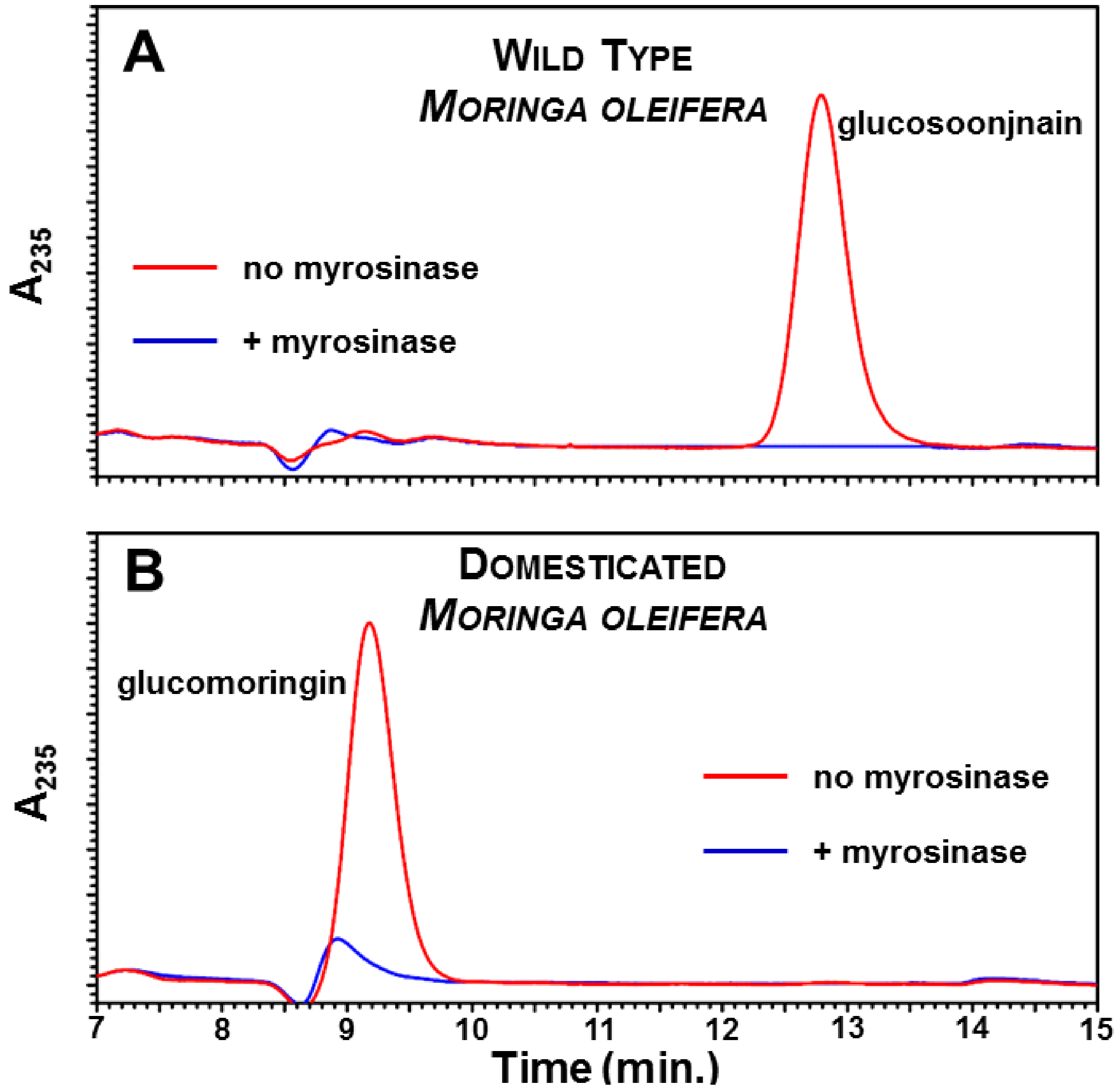

Supplement: Supplementary file 1 — Supplemental Figure S1 [file 41598_2018_26059_MOESM1_ESM.pdf]
